# Narcissistic traits and emotional dependence on large language models among adolescents

**DOI:** 10.3389/fpsyt.2026.1958023

**Published:** 2026-10-09

**Authors:** Yihang Zhao, Mengyue Xu, Ning Wang, Yang Liu, Hong Wang

**Affiliations:** 1 Wuhan Sports University, Wuhan, China; 2 Huangshan University, Huangshan, China; 3 Chengdu Vocational University of the Arts, Chengdu, China; 4 Jishou University, Jishou, China; 5 Fuyang Institute of Technology, Fuyang, China

**Keywords:** adolescents, anxiety, emotional dependence, large language models, narcissistic traits, physical activity

## Abstract

**Background:**

This study examined associations between narcissistic traits and emotional dependence on large language models (LLMs) among adolescents, focusing on anxiety and physical activity.

**Methods:**

A multistage stratified sample of 1622 Chinese junior high school students was surveyed in November 2025. Narcissistic traits, anxiety, LLM emotional dependence, and physical activity were assessed using the FFNI-SF, GAD-2, an adapted five-item LLM emotional dependence measure, and PARS-3, respectively. Pearson correlations and PROCESS Models 4 and 7 with 5,000 bootstrap resamples were conducted, with Johnson–Neyman analysis used to probe the interaction.

**Results:**

Narcissistic traits correlated positively with anxiety (r=0.118, P<0.001) and LLM emotional dependence (r=0.301, P<0.001), while anxiety correlated positively with LLM emotional dependence (r=0.146, P<0.001). Physical activity correlated negatively with anxiety (r=−0.119, P<0.001) and LLM emotional dependence (r=−0.075, P = 0.002). Adjusting for grade and left-behind status, anxiety showed a small indirect association between narcissistic traits and LLM emotional dependence (Effect=0.011, 95% CI [0.004, 0.020]). The narcissistic traits × physical activity interaction was significant (β=−0.056, P = 0.015), with the narcissism–anxiety association significant below 0.73 SD of physical activity. The moderated mediation index was not significant (Index=−0.0061, 95% bootstrap CI [−0.0130, 0.0001]).

**Conclusion:**

Narcissistic traits were positively associated with adolescent LLM emotional dependence, with a small cross-sectional indirect association through anxiety. Physical activity moderated the narcissism–anxiety association, although overall moderated mediation was not supported.

## 1 Introduction

With the rapid advancement of generative artificial intelligence technology, large language models (LLMs) have gradually evolved from traditional information processing tools into a new type of digital medium characterized by social interaction properties. Through capabilities such as natural language generation, contextual understanding, and sustained interaction, LLMs can provide feedback experiences resembling interpersonal communication and foster a certain degree of parasocial interaction (1). Previous research has found that some adolescents, through prolonged engagement with AI chatbots, may project their emotional needs onto technological objects and form psychological connections akin to human–AI attachment (2). Although this novel interaction mode can offer immediate companionship and emotional support, excessive dependence may undermine active participation in real-world social interactions and is associated with psychological adaptation issues such as social withdrawal and altered perceptions of reality (3). Therefore, identifying the key psychological factors and processes associated with adolescent LLM emotional dependence is of considerable importance for understanding emerging human–AI relationship patterns and promoting healthy use of artificial intelligence.

LLM emotional dependence refers to the persistent emotional connection and psychological attachment that individuals develop through their use of generative artificial intelligence, primarily manifested as a high endorsement of the value of AI interactions, discomfort upon communication disruption, and a continuous need to maintain interaction (4). As a personality factor associated with interpersonal interaction patterns, narcissistic traits may play a significant role in patterns of LLM emotional dependence. Narcissistic traits are typically characterized by an emphasis on self-importance, a persistent need for external attention, and self-centered tendencies in interpersonal relationships (5). According to the vulnerability-grandiosity dual-dimension model of narcissism, individuals with higher narcissistic traits may vary in their sensitivity to external evaluation; however, the present study examines narcissistic traits at the overall construct rather than distinguishing vulnerable and grandiose dimensions. Higher overall narcissistic traits may be associated with challenges in interpersonal relationships (6). Furthermore, attachment theory and object relations theory suggest that individuals with higher narcissistic tendencies may prefer interaction environments with greater controllability and lower evaluation risk, to satisfy their needs for self-worth maintenance and emotional support (7, 8). Compared with real interpersonal interactions, LLMs offer characteristics such as immediate responsiveness, high availability, and lower social evaluation pressure. These interaction features may provide adolescents with higher narcissistic tendencies a safer and more controllable emotional communication environment, making it easier for them to turn to AI as a source of emotional support. Consequently, narcissistic traits may be positively associated with adolescent LLM emotional dependence, such that individuals with higher narcissistic traits may exhibit greater degrees of LLM emotional dependence. Previous evidence on adolescent-AI emotional bonds converges in showing that anthropomorphic chatbots can elicit parasocial processes and emotional reliance in users (2), and that excessive reliance on AI companionship is associated with social withdrawal and psychological maladaptation (13). However, two critical gaps remain. First, although theoretical accounts implicate personality dispositions, almost no empirical study has tested whether narcissistic traits—characterized by validation-seeking and evaluation-sensitive self-regulation—are associated with adolescent LLM emotional dependence. Second, no study has examined whether modifiable health-behavior resources may be associated with attenuation of this personality-linked risk. It should also be noted that LLM emotional dependence is conceptually distinct from several related constructs: emotional dependence refers to a persistent need to maintain an affective bond within interpersonal relationships (21); human–AI attachment describes parasocial bonding with an AI agent that may occur without functional impairment (2); and psychological dependence and problematic AI system use emphasize behavioral components such as loss of control, withdrawal, and interference with daily functioning. In the present study, LLM emotional dependence denotes the persistent emotional reliance that adolescents place on interactions with LLMs, characterized by high endorsement of the value of AI interaction, discomfort when interaction is disrupted, and a continuous need to maintain interaction.

In the association between narcissistic traits and LLM emotional dependence, anxiety may constitute an important psychological pathway. The personality stress model and emotion regulation theory posit that individuals with higher narcissistic tendencies are typically more attentive to self-worth maintenance and more sensitive to external evaluation and interpersonal feedback; when real interactions fail to meet their self-confirmation needs, they may experience more intense negative emotional responses (9). Previous research has demonstrated that narcissistic traits, assessed at the overall trait level, are closely associated with emotion regulation difficulties and internalizing psychological problems, with affected individuals more likely to employ avoidant or compensatory strategies to cope with psychological stress (10). Anxiety is not only associated with greater psychological burden but may also be associated with the pursuit of more accessible and low-risk emotion regulation strategies (11). According to the Compensatory Internet Use Theory, when individuals face unmet psychological needs or heightened negative emotional experiences in real life, digital media may serve as an important alternative channel for alleviating psychological distress (12). Given that LLMs possess interactive features such as immediate responsiveness, high availability, and low social evaluation pressure, anxious individuals may be more inclined to obtain emotional support and psychological comfort through AI interactions, thereby increasing their tendency toward emotional dependence (13). Therefore, narcissistic traits may be further associated with adolescent LLM emotional dependence through the enhancement of anxiety experiences.

However, the process by which narcissistic traits are linked to anxiety experiences may be influenced by individuals’ psychological resources. Physical activity (PA), as an important health behavior, has been widely demonstrated to be closely associated with emotion regulation, self-evaluation, and psychological adaptation (14, 15). Theoretical accounts that separate narcissism from self-esteem hold that narcissism does not reflect stable positive self-esteem but rather an ongoing need for external validation and self-worth maintenance (16). Regular participation in physical activity is associated with enhanced body self-perception, self-efficacy, and positive emotional experiences, providing individuals with a source of self-evaluation grounded in genuine competence development, which may relate to strengthened psychological regulation resources (17). For adolescents with higher narcissistic traits, richer psychological resources may be associated with a lower likelihood of anxiety responses associated with threats to self-evaluation, thus corresponding to a weaker positive association between narcissistic traits and anxiety. Existing research has shown that physical activity is associated with emotion regulation capacity and psychological adaptation in adolescents (18), and may be associated with attenuation of personality-related risk at the bivariate level (17, 18). For narcissistic adolescents, whose self-worth depends heavily on external validation, the competence-based self-evaluation fostered by regular physical activity offers a functional alternative to the immediate but low-cost validation obtained from LLMs; it thereby targets the entry point of the dependence pathway (the narcissism-to-anxiety association) rather than merely reducing anxiety in general. Moreover, unlike screen-based AI interaction, physical activity is typically embedded in offline social contexts and can directly counteract the social withdrawal through which anxious individuals gravitate toward digital emotional support.

In summary, the overall objective of this study was to examine the association between narcissistic traits and emotional dependence on large language models (LLMs) among Chinese junior high school students and to clarify the roles of anxiety and physical activity in this association. Specifically, we examined whether anxiety accounted for an indirect association between narcissistic traits and LLM emotional dependence and whether physical activity moderated the association between narcissistic traits and anxiety. Accordingly, a moderated mediation model was proposed (**Figure 1**), with anxiety as the mediator and physical activity as the moderator. Based on this framework, the following hypotheses were formulated:

**Figure 1:**
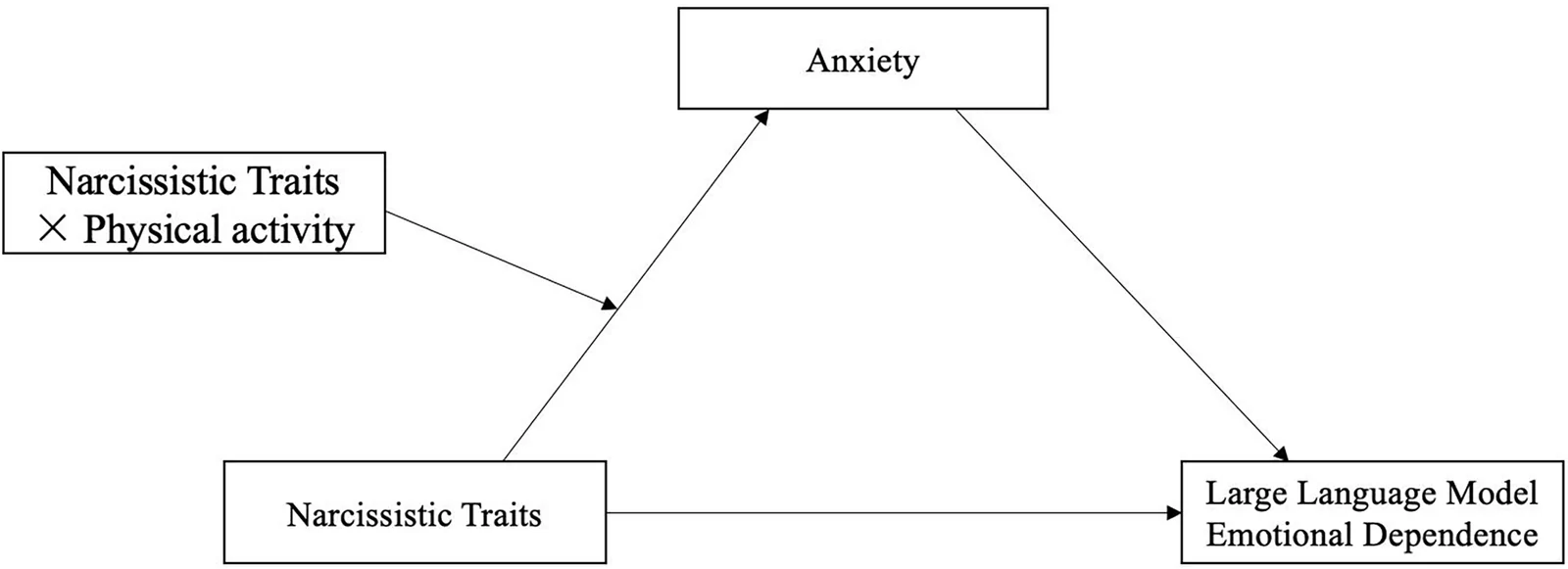
Hypothesized model.

H1: Narcissistic traits are positively associated with LLM emotional dependence;
H2: Anxiety accounts for a small indirect association in the relationship between narcissistic traits and LLM emotional dependence;
H3: Physical activity moderates the association between narcissistic traits and anxiety.

## 2 Methods

### 2.1 Participants

The study employed a multi-stage stratified sampling method to recruit participants. In each of the four study areas, one ordinary junior high school was selected, and two complete classes were randomly selected from each of the seventh, eighth, and ninth grades at each school, totaling 24 classes. All eligible students were invited to participate. Data collection was conducted in November 2025, and the questionnaires were distributed and collected in the same class on the same day. All 1,857 questionnaires were returned. After data quality screening, 235 invalid questionnaires were excluded, and 1,622 valid questionnaires were retained, with a valid questionnaire rate of 87.35%. The participants were aged between 10 and 19 years, with an average age of 14.64 years (standard deviation = 1.65). Participation was voluntary and anonymous. This study has been approved by the Medical Ethics Committee of Wuhan Sports University (Approval Number: 2024048). One week before data collection, written informed consent was obtained from the legal guardians of all participants, and written consent was also obtained from the adolescents themselves. Participants were informed that they could refuse to participate or withdraw from the study at any time without any penalty. The demographic characteristics of the sample are presented in **Table 1**.

**Table 1:** Demographic characteristics of the study sample (N = 1,622).

Sex	Boys	797	49.14
	Girls	825	50.86
Grade	Grade 7	536	33.05
	Grade 8	542	33.42
	Grade 9	544	33.54
Only-child status	Only child	823	50.74
	Non-only child	799	49.26
Left-behind status	Left-behind	456	28.11
	Non-left-behind	1166	71.89

Left-behind status was operationalized through a single demographic item: “During your childhood, have you ever experienced a period when your parents were absent for less than six months or longer?”.

### 2.2 Measures

#### 2.2.1 Narcissistic traits

Narcissistic traits were assessed using the 15-item Chinese brief version of the Five-Factor Narcissism Inventory (FFNI) validated by Chen et al. (2024) (19). The measure is grounded in the Five-Factor Model of personality and captures the heterogeneous structure of narcissism. In addition to an overall narcissism score, the items can be organized into three higher-order components: agentic extraversion, reflecting assertiveness, grandiose self-presentation, and achievement-oriented self-enhancement; antagonism, reflecting entitlement, exploitativeness, interpersonal callousness, and antagonistic self-regulation; and narcissistic neuroticism, reflecting evaluation sensitivity, shame, and negative emotionality. However, because the 15-item short form does not allow reliable separation of vulnerable and grandiose dimensions, the present study used the total FFNI-SF score as the primary index rather than making vulnerable/grandiose dimensional distinctions. Each item was rated on a 5-point Likert scale ranging from 1(strongly disagree) to 5(strongly agree).

#### 2.2.2 Emotional dependence on LLMs

Emotional dependence on LLMs was assessed using five items adapted from the Affective Dependence Scale developed by Sirvent-Ruiz et al. and the Rapport–Expectation with a Robot Scale developed by Nomura and Kanda (20, 21). Items reflecting emotional reliance, relational need, and perceived emotional connection were selected and recontextualized by replacing the original interpersonal or human–robot referents with LLM-related interaction contexts (22). The adaptation was intended to preserve the relational and emotional content of the source items while changing the interaction target to LLMs. Accordingly, the measure assessed generalized emotional dependence on LLM-based interaction rather than dependence on a specific commercial platform. All five items were rated on a 5-point Likert scale ranging from 1 (strongly disagree) to 5 (strongly agree), yielding a total score of 5–25, with higher scores indicating stronger emotional dependence on LLMs. In the full sample, the measure demonstrated excellent internal consistency (Cronbach’s α = .948), with corrected item–total correlations ranging from 0.801 to 0.884. Given the contextual adaptation of the measure, its factorial structure was further examined using independent exploratory and confirmatory subsamples. The valid sample (N = 1,622) was randomly divided into two equal subsamples (n=811 each). In the exploratory subsample, principal axis factoring was used for factor extraction, and the number of factors to retain was determined by parallel analysis. The data showed adequate factorability (KMO = 0.901; Bartlett’s χ²(10) = 3923.63, P<0.001). Parallel analysis supported a single-factor solution: the first empirical eigenvalue (4.135) exceeded the corresponding 95th-percentile random eigenvalue (1.145), whereas the second empirical eigenvalue (0.319) was below its random criterion (1.073). Because only one factor was retained, rotation was not applied, as factor rotation is only meaningful when two or more factors are extracted. The retained factor accounted for 78.44% of the variance, with factor loadings ranging from.840 to.910. The one-factor structure was subsequently examined using CFA in the independent validation subsample (n = 811). Standardized factor loadings ranged from 0.809 to 0.911. The model demonstrated acceptable fit, χ²(5) = 23.18, p < 0.001, CFI = 0.987, TLI = 0.974, RMSEA = 0.067 (90% CI [0.041, 0.096]), and SRMR = 0.025. Composite reliability (CR = 0.949) and average variance extracted (AVE = 0.788) exceeded conventional criteria, providing preliminary support for the unidimensional structure, construct reliability, and convergent validity of the adapted five-item measure.

#### 2.2.3 Anxiety

Anxiety symptoms were assessed using the two-item Generalized Anxiety Disorder scale (GAD-2), which comprises the two core anxiety items from the GAD-7 and has been widely used as an ultra-brief indicator of anxiety symptom severity (23). Each item assesses symptom frequency during the previous two weeks on a 4-point scale ranging from 0 (not at all) to 3 (nearly every day), yielding a total score of 0–6, with higher scores indicating greater anxiety symptom severity. The GAD-2 was selected because the present study involved a large school-based survey containing multiple psychological and behavioral measures, and a brief instrument helped reduce respondent burden while retaining coverage of the core symptoms of generalized anxiety (35). Previous validation studies have supported the screening utility of the GAD-2, including the commonly used cutoff of ≥3 for clinically relevant anxiety symptoms (36). In the present sample, the two items showed good internal consistency (Cronbach’s α = .869; inter-item r = .768).

#### 2.2.4 Physical activity

Physical activity was quantified using the Physical Activity Rating Scale (PARS-3) developed by Liang (24). The scale assesses habitual physical activity in terms of exercise intensity, session duration, and weekly frequency. The total physical activity score is calculated as Intensity × (Duration − 1) × Frequency, yielding a possible range of 0–100, with higher scores indicating greater physical activity. Because the PARS-3 score is a formative composite index derived multiplicatively from these three components, internal consistency reliability such as Cronbach’s α is not an appropriate psychometric property and was therefore not calculated or reported.

### 2.3 Quality control

To ensure data quality and consistency, all researchers involved in data collection received standardized training before the formal survey. Data collection was conducted in November 2025 using paper-and-pencil questionnaires administered collectively in classrooms. Questionnaires were distributed and collected on site during the same class session, with completion requiring approximately 20–30 min; no mobile phones, computers, or web-based survey platforms were used. Trained researchers provided standardized instructions, and participants completed the questionnaires independently. Anonymity, confidentiality, voluntary participation, and the absence of right or wrong answers were emphasized to minimize social desirability and potential common method bias. A total of 1,857 questionnaires were distributed and all were collected on site. After collection, all questionnaires were screened for data quality. Questionnaires meeting one or more of the predefined exclusion criteria—substantial missing responses, obvious patterned responding, or logical inconsistencies—were excluded from further analysis. A total of 235 questionnaires were excluded, leaving 1,622 valid questionnaires for the final analyses, corresponding to a valid questionnaire rate of 87.35%. Data entry was independently checked, and missing values and outliers in the retained dataset were further screened before statistical analysis.

### 2.4 Statistical analysis

Statistical analyses were performed using IBM SPSS Statistics version 27.0 and R version 4.6.0. All statistical tests were two-tailed, with the significance level set at P<0.05. Continuous variables were initially examined for distributional characteristics using skewness and kurtosis. Following Kim’s criteria, absolute skewness values < 2 and absolute kurtosis values < 7 were considered indicative of acceptable approximate normality (25). Because the study variables were assessed using self-report measures, potential common method bias (CMB) was preliminarily examined using Harman’s single-factor test. All measurement items were entered simultaneously into an unrotated principal component analysis to determine whether a dominant general component accounted for a substantial proportion of the total variance. This procedure was used solely as a preliminary diagnostic of potential common method variance and was not used to determine the dimensionality or construct validity of the individual measures (26). Given the contextual adaptation of the five-item measure of emotional dependence on LLMs, its factorial structure was evaluated separately from the CMB analysis. The valid sample (N = 1,622) was randomly divided into two independent subsamples of equal size (n=811 each). In the exploratory subsample, principal axis factoring was used for factor extraction, and parallel analysis was used to determine the number of factors to retain. Because parallel analysis supported a single-factor solution, factor rotation was not applicable. The resulting factor structure was subsequently cross-validated using confirmatory factor analysis (CFA) in the independent validation subsample. CFA was conducted in R using the *lavaan* package, and model fit was evaluated using the chi-square statistic, comparative fit index (CFI), Tucker–Lewis index (TLI), root mean square error of approximation (RMSEA), and standardized root mean square residual (SRMR). Composite reliability (CR) and average variance extracted (AVE) were additionally calculated to evaluate construct reliability and convergent validity. Descriptive statistics and Pearson correlation analyses were then conducted to characterize the study variables and examine their bivariate associations. Prior to the primary model analyses, preliminary demographic comparisons were performed to examine variation in LLM emotional dependence across sex, grade, only-child status, and left-behind status. Independent-samples *t* tests were used for binary demographic variables, whereas one-way analysis of variance (ANOVA) was used for grade. These analyses were exploratory and were conducted to inform covariate adjustment rather than to address independent substantive research objectives. Based on the preliminary demographic analyses, grade and left-behind status were retained as covariates in the subsequent mediation and moderated mediation models. After adjustment for these demographic covariates, mediation and moderated mediation analyses were conducted using Hayes’s PROCESS macro version 4.1 for SPSS. PROCESS Model 4 was used to examine the indirect association between narcissistic traits and LLM emotional dependence through anxiety symptoms. PROCESS Model 7 was subsequently used to examine whether physical activity moderated the association between narcissistic traits and anxiety symptoms. The index of moderated mediation and conditional indirect associations at low (−1 SD), mean, and high (+1 SD) levels of physical activity were estimated using 5,000 bootstrap resamples, with effects considered statistically significant when the 95% bootstrap confidence interval did not include zero. The Johnson–Neyman technique was used to identify regions of significance for the narcissism–anxiety association across the observed range of physical activity. Given the positively skewed distribution of PARS-3 scores, an additional sensitivity analysis was conducted by categorizing physical activity according to conventional PARS-3 criteria (low ≤19, moderate 20–42, and high ≥43) and re-estimating the narcissistic traits × physical activity interaction.

## 3 Results

### 3.1 Common method bias

Because all study variables were assessed using self-report measures, the potential influence of common method bias (CMB) was examined using Harman’s single-factor test as a preliminary diagnostic. All items measuring narcissistic traits, LLM emotional dependence, anxiety symptoms, and physical activity were entered simultaneously into an unrotated principal component analysis. Six components with eigenvalues greater than 1.0 were extracted, and the first component accounted for 21.924% of the total variance, which was below the commonly used 40% criterion. Thus, no single component accounted for a predominant proportion of the total variance.

### 3.2 Correlation analysis

Skewness and kurtosis were −0.135 and 0.748 for narcissistic traits, 0.805 and −0.011 for anxiety symptoms, 1.707 and 2.622 for physical activity, and 0.116 and −1.395 for LLM emotional dependence, respectively. Descriptive analysis showed that GAD-2 scores ranged from 0 to 6, with a mean of 1.96 (SD = 1.74), a median of 2.00, and an interquartile range of 0–3. A total of 412 participants (25.40%) scored ≥3. Pearson correlation analysis (**Table 2**) showed that narcissistic traits were positively correlated with anxiety symptoms (r=0.118, P<0.001) and emotional dependence on LLMs (r=0.301, P<0.001). Anxiety symptoms were positively correlated with emotional dependence on LLMs (r=0.146, P<0.001), whereas physical activity was negatively correlated with anxiety symptoms (r=−0.119, P<0.001) and emotional dependence on LLMs (r=−0.075, P<0.01). Narcissistic traits were not significantly associated with physical activity (r=−0.030, P = 0.232). Overall, the observed associations were small in magnitude, with the exception of the comparatively stronger association between narcissistic traits and LLM emotional dependence (r=0.301). The corresponding squared correlations indicated that the significant bivariate associations shared approximately 0.6%–9.1% of their variance.

**Table 2:** Correlation analysis of study variables.

Variable	M	SD	1	2	3	4
1. Narcissistic traits	40.95	9.05	–			
2. Anxiety	1.96	1.74	0.118^***^	–		
3. Physical activity	20.34	23.83	-0.030	-0.119^***^	–	
4. LLM emotional dependence	12.27	6.24	0.301^***^	0.146^***^	-0.075^**^	–

*p < 0.05, **p < 0.01, ***p < 0.001. Same hereinafter.

### 3.3 Preliminary demographic analyses

Preliminary demographic comparisons were conducted (**Table 3**) to examine variations in LLM emotional dependence across selected demographic characteristics and to inform covariate adjustment in the subsequent analyses. No significant differences in LLM emotional dependence were observed by sex or only-child status (P>0.05). Left-behind adolescents reported slightly higher levels of LLM emotional dependence than non-left-behind adolescents (t=2.785, P = 0.005, Cohen’s d=0.151). A small grade-related variation was also observed (F = 3.005, P = 0.050, η²=0.004). Based on these preliminary analyses, grade and left-behind status were retained as covariates in the subsequent mediation and moderated mediation models.

**Table 3:** Preliminary demographic comparisons of LLM emotional dependence.

Demographic variable	Group	M ± SD	T/F	P	Effect size
Sex	Male	12.15 ± 6.40	t=-0.754	0.451	d=-0.037
	Female	12.39 ± 6.08			
Only-child status	Only child	12.68 ± 6.18	t=1.439	0.150	d=0.085
	Non-only child	12.15 ± 6.25			
Left-behind status	Left-behind	12.93 ± 6.20	t=2.785	0.005	d=0.151
	Non-left-behind	11.99 ± 6.23			
Grade	Grade 7	11.34 ± 6.27	F=3.005	0.050	η²=0.004
	Grade 8	12.42 ± 6.17			
	Grade 9	12.45 ± 6.33			

Cohen’s d and η² denote effect sizes for t-tests and ANOVA, respectively.

### 3.4 Mediating role of anxiety

Hayes’s PROCESS Model 4 was used to examine the indirect association between narcissistic traits and LLM emotional dependence through anxiety symptoms, with grade and left-behind status included as covariates. Narcissistic traits were positively associated with anxiety symptoms (β = 0.098, SE = 0.024, P < 0.001), and anxiety symptoms were positively associated with LLM emotional dependence (β = 0.110, SE = 0.024, P < 0.001). After accounting for anxiety symptoms, the direct association between narcissistic traits and LLM emotional dependence remained statistically significant (β = 0.288, SE = 0.024, P < 0.001). The bootstrap analysis indicated a statistically significant but small indirect association through anxiety (Effect = 0.011, SE = 0.004, 95% CI [0.004, 0.020]; **Table 4**).

**Table 4:** Mediation analysis of anxiety between narcissistic traits and LLM emotional dependence.

Outcome variable	Predictor	β[95%CI]	SE	t	R²	F
Anxiety	Narcissistic traits	0.098 [0.050, 0.146]	0.024	4.015^***^	0.044	24.747^***^
	Grade	0.156 [0.107, 0.204]	0.024	6.356^***^		
	Left-behind status	-0.069 [-0.117, -0.021]	0.024	-2.815^**^		
LLM emotional dependence	Narcissistic traits	0.288 [0.241, 0.334]	0.024	12.089^***^	0.106	47.859^***^
	Anxiety	0.110 [0.063, 0.157]	0.024	4.561^***^		
	Grade	-0.010 [-0.057, 0.038]	0.024	-0.400		
	Left-behind status	-0.053 [-0.100, -0.007]	0.024	-2.256^*^		

β = standardized coefficient. Same hereinafter.

### 3.5 Moderating role of physical activity

To further examine the moderating role of physical activity in the process by which narcissistic traits influence LLM emotional dependence, Hayes’s PROCESS Model 7 was used to construct a moderated mediation model (**Figure 2**), with left-behind status and grade included as covariates. The results showed that, after controlling for covariates, narcissistic traits significantly and positively associated with anxiety (β=0.098, SE = 0.024, P<0.001), and physical activity significantly and negatively associated with anxiety (β=−0.096, SE = 0.025, P<0.001). Furthermore, the interaction term between narcissistic traits and physical activity had a significant negative predictive effect on anxiety (β=−0.056, SE = 0.023, P = 0.015), indicating that physical activity significantly moderated the relationship between narcissistic traits and anxiety (**Table 5**).

**Figure 2:**
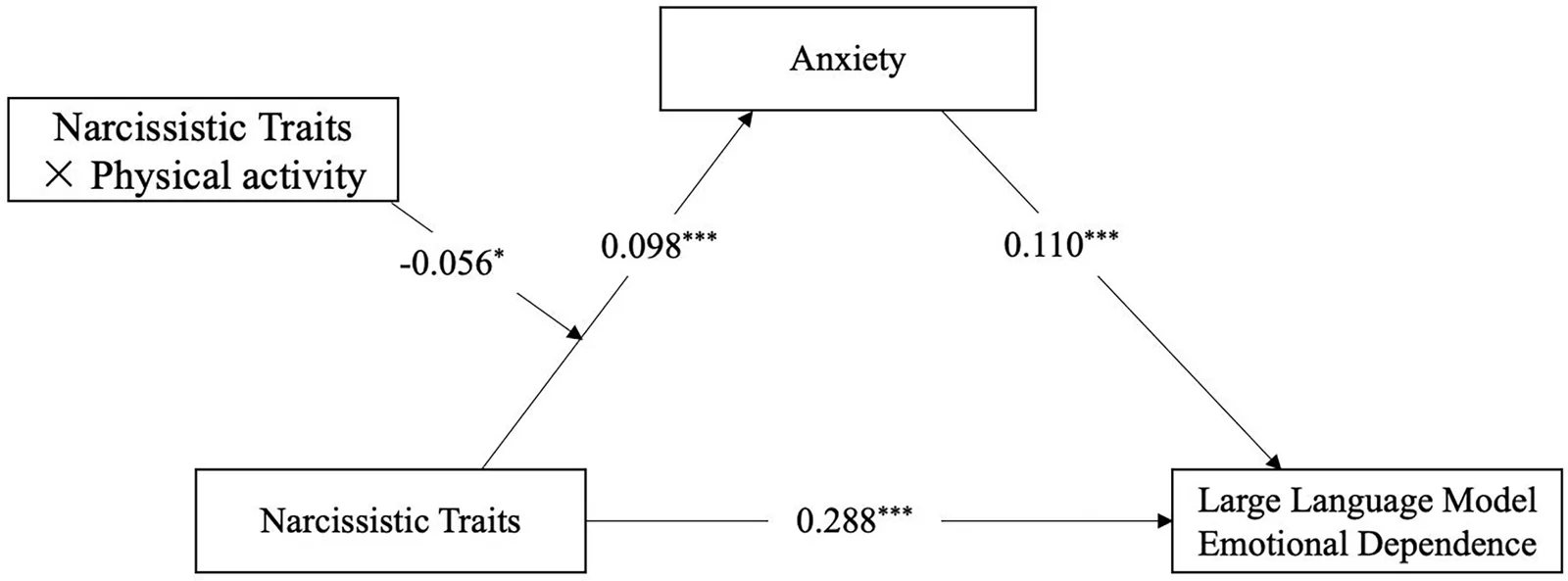
Moderated mediation model.

**Table 5:** Moderated mediation model test results.

Outcome variable	Predictor	β [95%CI]	SE	t	R²	F
Anxiety	Narcissistic traits	0.098 [0.050, 0.146]	0.024	4.027^***^	0.055	18.932^***^
	Physical activity	-0.096 [-0.144, -0.048]	0.025	-3.899^***^		
	Narcissistic traits × Physical activity	-0.056 [-0.100, -0.011]	0.023	-2.429^*^		
	Left-behind status	-0.063 [-0.111, -0.016]	0.024	-2.611^**^		
	Grade	0.141 [0.093, 0.190]	0.025	5.721^***^		
LLM emotional dependence	Narcissistic traits	0.288 [0.241, 0.334]	0.024	12.089^***^	0.106	47.859^***^
	Anxiety	0.110 [0.063, 0.157]	0.024	4.561^***^		
	Left-behind status	-0.053 [-0.100, -0.007]	0.024	-2.256^*^		
	Grade	-0.010 [-0.057, 0.038]	0.024	-0.400		

β=standardized coefficient. Same hereinafter. The index of moderated mediation was -0.0061 (BootSE = 0.0033, 95% bootstrap CI [-0.0130, 0.0001]). Conditional indirect associations at -1 SD, mean, and +1 SD of physical activity were 0.0168 (95% CI [0.006, 0.030]), 0.0108 (95% CI [0.004, 0.021]), and 0.0047 (95% CI [-0.003, 0.015]), respectively.

To further characterize the significant interaction, the Johnson–Neyman technique was used to identify the region of significance (**Figure 3**) for the conditional association between narcissistic traits and anxiety symptoms. Standardized physical activity values observed in the sample ranged from −0.85 to 3.34. The Johnson–Neyman analysis identified critical values of 0.73 and 9.36 standard deviations. Because the upper critical value of 9.36 was outside the observed range of physical activity, only the lower boundary was empirically relevant. Within the observed range, the conditional association between narcissistic traits and anxiety symptoms was statistically significant when physical activity was below 0.73 SD, whereas its 95% confidence interval included zero when physical activity ranged from 0.73 to 3.34 SD. The lower Johnson–Neyman boundary corresponded to a raw PARS-3 score of 37.71; 272 participants (16.77%) had observed PARS-3 scores above this boundary. Thus, within the range represented in the sample, the positive narcissism–anxiety association was statistically distinguishable from zero only at physical activity values below the Johnson–Neyman boundary. The value of 37.71 represents a sample-specific region-of-significance boundary and should not be interpreted as a clinical or intervention threshold. The index of moderated mediation was −0.0061 (BootSE=0.0033, 95% bootstrap CI [−0.0130, 0.0001]). Because the bootstrap confidence interval included zero, the overall index of moderated mediation was not statistically significant. The conditional indirect association between narcissistic traits and LLM emotional dependence through anxiety symptoms was 0.0168 (95% bootstrap CI [0.006, 0.030]) at physical activity of −1 SD, 0.0108 (95% bootstrap CI [0.004, 0.021]) at the mean level of physical activity, and 0.0047 (95% bootstrap CI [−0.003, 0.015]) at +1SD. Therefore, the present data did not provide statistically conclusive evidence that the indirect association varied systematically as a function of physical activity.

**Figure 3:**
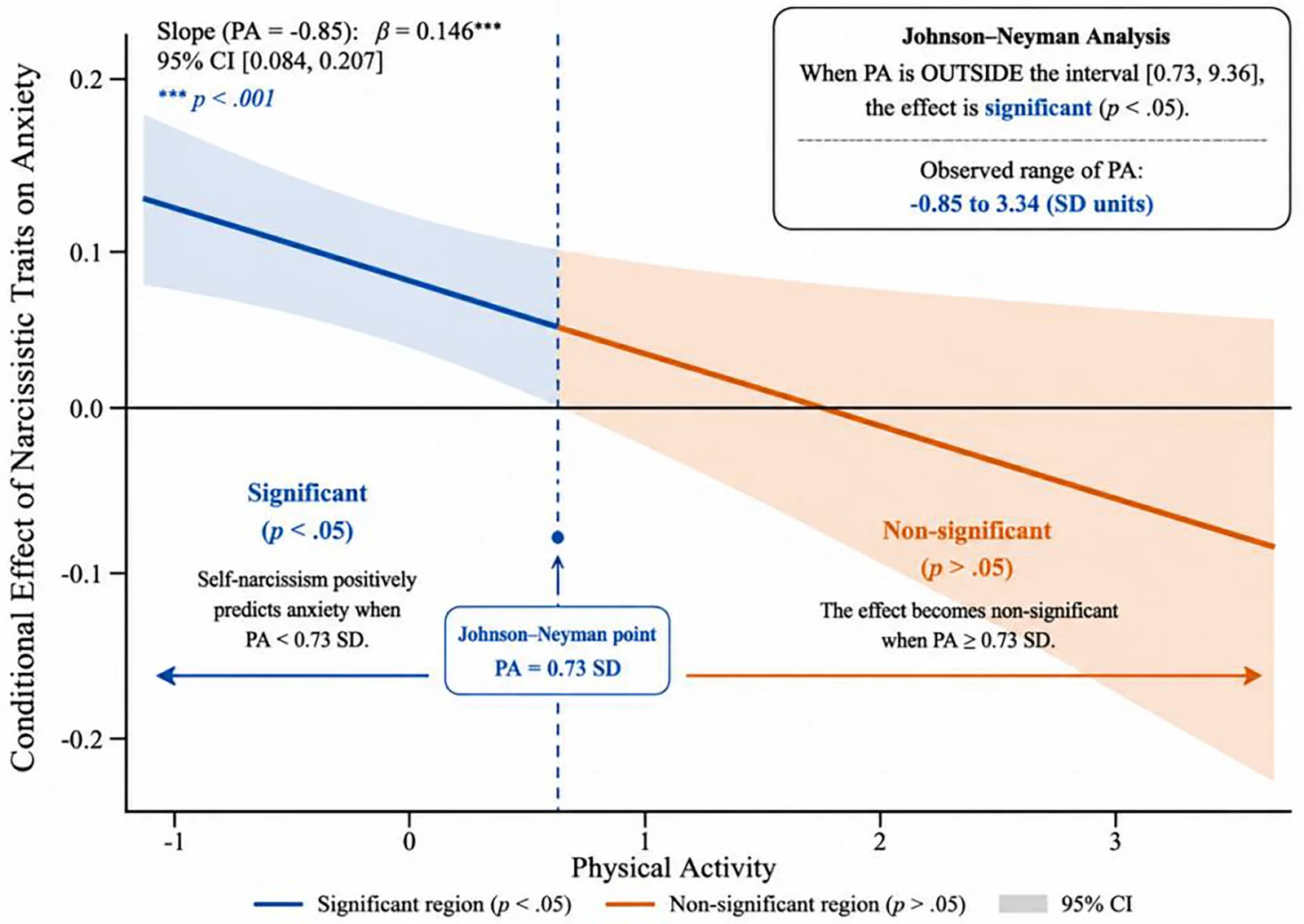
Johnson–Neyman plot of the moderating effect of physical activity on the relationship between narcissistic traits and anxiety.

Given the positively skewed distribution of PARS-3 scores, a supplementary sensitivity analysis was conducted using conventional PARS-3 activity categories while retaining the continuous specification as the primary analysis. Participants were classified as having low (≤19; n=1,041, 64.18%), moderate (20–42; n= 343, 21.15%), or high (≥43; n=238, 14.67%) physical activity. The overall narcissistic traits × physical activity-category interaction was statistically significant, F(2, 1614)=4.354, P = 0.013. The association between narcissistic traits and anxiety symptoms was significant in the low-activity category (β=0.106, P<0.001) and the moderate-activity category (β=0.188, P<0.001), but not in the high-activity category (β=−0.054, P = 0.393). Thus, the sensitivity analysis supported heterogeneity in the narcissism–anxiety association across physical activity levels.

## 4 Discussion

Drawing on a theoretical framework of the interactive effects of personality traits, emotional states, and health behaviors, this study constructed a moderated mediation model of physical activity–moderated narcissistic traits, anxiety, and emotional dependence on large language models (LLMs), with the aim of examining the psychological processes associated with adolescent LLM emotional dependence. The findings indicated that narcissistic traits were significantly and positively associated with LLM emotional dependence, suggesting that individual personality characteristics may be closely linked to patterns of emotional interaction with artificial intelligence. Further analysis revealed a small but significant indirect association through anxiety between narcissistic traits and LLM emotional dependence, indicating that negative emotional experiences may represent one psychological process connecting personality characteristics to AI emotional dependence. Moreover, physical activity significantly moderated the relationship between narcissistic traits and anxiety, with higher levels of physical activity attenuating the positive association between narcissistic traits and anxiety. Overall, this study extends research on adolescent LLM emotional dependence from three perspectives—personality risk factors, emotion regulation mechanisms, and positive health behavior resources—providing new empirical evidence for understanding adolescent psychological adaptation in the context of generative artificial intelligence.

This study found that narcissistic traits were significantly and positively associated with LLM emotional dependence, indicating that personality factors may be involved in shaping adolescents’ psychological tendencies toward interaction with artificial intelligence. Individuals with higher narcissistic traits typically exhibit stronger self-focused tendencies and needs for external validation, with their sense of self-worth partly dependent on external feedback and social evaluation (4, 5). In real interpersonal interactions, individuals must navigate complex interpersonal feedback and potential evaluation pressure, whereas the high availability, immediate responsiveness, and lower social evaluation risk characteristic of LLMs may provide some adolescents with a safer and more controllable interaction environment, making it easier for them to turn to LLMs as important sources of emotional expression, self-confirmation, or psychological support (27, 28). From a psychological process perspective, LLMs do not simply replace real interpersonal relationships but may become novel interaction objects that satisfy specific psychological needs. For adolescents with higher narcissistic traits, the AI interaction environment may reduce the evaluation risks inherent in real relationships, making it easier to obtain positive feedback and emotional responses. Therefore, the present findings suggest that LLM emotional dependence is associated not only with technology availability but also with individuals’ own personality characteristics and psychological needs.

Further analysis revealed a small but significant indirect association through anxiety between narcissistic traits and LLM emotional dependence (approximately 3.7% of the total effect). However, the cross-sectional design precludes causal inferences, and this small effect size does not support characterizing anxiety as a dominant psychological transmission mechanism. Individuals with higher narcissistic tendencies, due to their greater concern with self-evaluation and external feedback, may exhibit higher levels of anxiety when facing interpersonal stress, self-worth threats, or social evaluation uncertainty (29). In anxious states, individuals typically have stronger emotion regulation needs and tend to seek rapid, low-cost, and easily accessible forms of emotional support (30). Because LLMs can provide immediate responsiveness and sustained interaction, they may serve as a supplementary channel for anxious individuals to regulate negative emotions, thereby being associated with higher levels of emotional dependence. This finding further indicates that LLM emotional dependence is not simply accounted for by personality traits directly but may be associated with the psychological process of emotional experience. From a compensatory use perspective, when individuals are in heightened anxiety states, their dependence on digital interaction resources may increase to obtain temporary emotional relief (11). The present findings further extend the applicability of this theory to the context of generative artificial intelligence, suggesting that individuals’ LLM emotional dependence may not arise solely from the attractiveness of the technology itself but is closely related to their real-world psychological needs and emotion regulation processes. A key feature distinguishing LLMs from traditional internet media is their capacity to provide continuous bidirectional communication experiences, which may strengthen the psychological connection between individuals and artificial intelligence, making LLMs more likely to become important objects in the emotion regulation process. The proportion of the total effect mediated by anxiety was approximately 3.7% (indirect effect=0.011; total effect=0.299), indicating that anxiety accounts for a small fraction of the narcissism–LLM emotional dependence association.

The study further found that physical activity significantly moderated the association between narcissistic traits and anxiety, indicating statistical moderation. Specifically, the Johnson–Neyman analysis showed that the positive association between narcissistic traits and anxiety was statistically significant when physical activity was below 0.73 SD, whereas the 95% confidence interval included zero when physical activity ranged from 0.73 to 3.34 SD. Thus, the narcissism–anxiety association was weaker at higher levels of physical activity within the observed range. However, given the cross-sectional design, this pattern should be interpreted as statistical moderation rather than evidence of a causal protective effect of physical activity. This finding suggests that physical activity may alter the strength of the association between personality risk factors and anxiety—that is, higher levels of physical activity may be associated with attenuation of the positive link between narcissistic traits and anxiety experiences. This finding further extends prior research perspectives on the mental health benefits of physical activity. Previous studies have primarily focused on the direct ameliorative effects of physical activity on negative emotions such as anxiety and depression, whereas the present study, from a risk-buffering perspective, found that physical activity may serve a buffering role—its influence may not necessarily manifest as directly reducing individuals’ emotional problem levels but rather as lowering the probability of risk factors translating into negative emotions when individuals possess higher psychological risk characteristics. This pattern is broadly consistent with theoretical accounts of stress moderation; however, given the cross-sectional design, it should be interpreted as an associative pattern rather than evidence of a causal stress-buffering mechanism (17, 18, 31, 32). Furthermore, unlike the individualized and screen-based nature of AI interactions, physical activity often occurs in real social contexts, providing participants with a sense of physical mastery, teamwork experiences, and authentic peer support. These positive interpersonal experiences may be associated with reduced psychological imbalance that narcissistic individuals experience in relation to real-world social setbacks. A plausible explanation is that physical activity, through associations with self-efficacy, positive bodily experiences, and increased opportunities for real social interaction (33, 34), is associated with adolescents developing a more stable self-evaluation system and reduced reliance on external feedback. J–N analysis further revealed the boundary conditions under which physical activity is associated with attenuation of the narcissism–anxiety association: This finding suggests that, in the context of generative artificial intelligence, the association between narcissistic traits and anxiety may vary according to physical activity levels; however, because all variables were measured concurrently, this pattern does not support promoting physical activity as an evidence-based intervention for reducing LLM emotional dependence.

In summary, this study constructed a moderated mediation model in which physical activity moderated the relationships among narcissistic traits, anxiety, and LLM emotional dependence, revealing the associations among personality characteristics, emotional factors, and health behaviors in relation to adolescent LLM emotional dependence. The findings showed that narcissistic traits were significantly and positively associated with LLM emotional dependence, anxiety was associated with a small indirect association, and physical activity corresponded to a weaker positive association between narcissistic traits and anxiety. These results suggest that adolescent LLM emotional dependence is not solely associated with technological factors but may be related to the combined association of individual psychological needs and behavioral resources. Several methodological features strengthen confidence in the present findings. First, the relatively large sample (N = 1,622) provided adequate statistical power for detecting associations of modest magnitude. Second, participants were recruited from schools across multiple regions, which increased sample heterogeneity, although the sample should not be considered nationally representative. Third, standardized researcher training and classroom-based administration helped maintain consistency in data collection procedures. Finally, the use of 5,000 bootstrap resamples for estimating indirect and conditional indirect effects reduced reliance on normality assumptions for the sampling distribution of these effects and provided more robust confidence-interval estimation. Together, these features enhance the methodological rigor of the study while not eliminating the limitations inherent in its cross-sectional and self-report design.

This study has several limitations. First, the cross-sectional design precludes conclusions regarding temporal precedence or causality; therefore, the indirect and moderating effects should be interpreted as cross-sectional statistical associations. Second, all focal variables were assessed using self-report measures, which may introduce common method and reporting biases. Actual LLM exposure, including platform, frequency, duration, and purpose of use, was not measured, limiting our ability to distinguish emotional dependence from differences in opportunities for LLM use. Third, although the sample was relatively large, participants were nested within classes and schools, and the primary analyses did not fully account for this clustered structure; consequently, standard errors for some estimates may be underestimated. Unfortunately, the de-identified data did not retain school- or class-level identifiers, so the intraclass correlation coefficient (ICC), design effect, and cluster-corrected standard errors could not be reliably estimated *post hoc*. Results with p-values close to the conventional significance threshold should be interpreted with particular caution. Fourth, covariates in the primary models (grade and left-behind status) were selected on the basis of their bivariate associations with the outcome in preliminary analyses. Selection based solely on bivariate significance does not account for the multivariate confounding structure and may not identify all relevant covariates; thus, the covariate set should be interpreted with caution. In addition, residual confounding remains possible because potentially relevant factors, such as depressive symptoms, academic stress, and perceived social support, were not assessed. Fifth, anxiety was measured using the brief GAD-2, and the adapted five-item measure of LLM emotional dependence requires further validation across independent samples and cultural contexts. Finally, the observed effect sizes were generally small, and the sample was restricted to Chinese junior high school students, limiting the generalizability of the findings. Future longitudinal, multi-method, and multi-site studies incorporating objective LLM-use indicators and more comprehensive psychological assessments are warranted.

## 5 Conclusion

This study, examining factors associated with adolescent LLM emotional dependence in the context of generative artificial intelligence, constructed a theoretical model of the interactive effects of personality traits, emotional mechanisms, and health behaviors, systematically examining the roles of narcissistic traits, anxiety, and physical activity in relation to LLM emotional dependence. The findings indicate that LLM emotional dependence is associated with multiple factors including personality dispositions and psychological processes, though effect sizes are generally small. Narcissistic traits show a small-to-medium positive association with LLM emotional dependence, with a small indirect association through anxiety (approximately 3.7% of the total effect) linking personality characteristics to tendencies for emotional interaction with artificial intelligence. Meanwhile, higher physical activity levels were associated with a weaker narcissism–anxiety association, though effect sizes are small for adolescents with psychological risk characteristics. This study further extends the explanatory paradigm of traditional technology dependence research, which has primarily focused on media characteristics and usage behaviors, by incorporating personality factors, emotional processes, and health behaviors into the research framework on generative artificial intelligence use, providing new empirical evidence for understanding the psychological processes through which adolescents relate to LLMs. From a practical perspective, the prevention of LLM emotional dependence should not be limited to technological restrictions but should also attend to adolescents’ psychological needs, emotion regulation capacity, and real-world social support resources.

## Data Availability

The raw data supporting the conclusions of this article will be made available by the authors, without undue reservation.
